# Population Genomics of an Anadromous Hilsa Shad *Tenualosa ilisha* Species across Its Diverse Migratory Habitats: Discrimination by Fine-Scale Local Adaptation

**DOI:** 10.3390/genes11010046

**Published:** 2019-12-30

**Authors:** Md Asaduzzaman, Yoji Igarashi, Md Abdul Wahab, Md Nahiduzzaman, Md Jalilur Rahman, Michael J. Phillips, Songqian Huang, Shuichi Asakawa, Md Moshiur Rahman, Li Lian Wong

**Affiliations:** 1 Department of Marine Bioresource Science, Faculty of Fisheries, Chattogram Veterinary and Animal Sciences University, Khulsi, Chattogram 4225, Bangladesh; 2 Department of Aquatic Bioscience, The University of Tokyo, 1-1-1 Yayoi, Bunkyo-ku, Tokyo 113-8657, Japan; aiga@mail.ecc.u-tokyo.ac.jp (Y.I.); huangsongqian0115@gmail.com (S.H.); asakawa@mail.ecc.u-tokyo.ac.jp (S.A.); 3 WorldFish, Bangladesh and South Asia Office, Banani, Dhaka 1213, Bangladesh; a.wahab@cgiar.org (M.A.W.); n.md@cgiar.org (M.N.); j.rahman@cgiar.org (M.J.R.); 4 WorldFish Headquarters, Jalan Batu Maung, Batu Muang, Penang 11960, Malaysia; m.phillips@cgiar.org; 5 Fisheries and Marine Resource Technology Discipline, Khulna University, Khulna 9208, Bangladesh; mrahmankufmrt@gmail.com; 6 Institute of Marine Biotechnology, Universiti Malaysia Terengganu, Kuala-Terengganu, Terengganu 21030, Malaysia

**Keywords:** population structure, conservation genetics, selection, anadromous fish, NextRAD sequencing, migratory fish

## Abstract

The migration of anadromous fish in heterogenic environments unceasingly imposes a selective pressure that results in genetic variation for local adaptation. However, discrimination of anadromous fish populations by fine-scale local adaptation is challenging because of their high rate of gene flow, highly connected divergent population, and large population size. Recent advances in next-generation sequencing (NGS) have expanded the prospects of defining the weakly structured population of anadromous fish. Therefore, we used NGS-based restriction site-associated DNA (NextRAD) techniques on 300 individuals of an anadromous Hilsa shad (*Tenualosa ilisha*) species, collected from nine strategic habitats, across their diverse migratory habitats, which include sea, estuary, and different freshwater rivers. The NextRAD technique successfully identified 15,453 single nucleotide polymorphism (SNP) loci. Outlier tests using the F_ST_ OutFLANK and pcadapt approaches identified 74 and 449 SNPs (49 SNPs being common), respectively, as putative adaptive loci under a divergent selection process. Our results, based on the different cluster analyses of these putatively adaptive loci, suggested that local adaptation has divided the Hilsa shad population into two genetically structured clusters, in which marine and estuarine collection sites were dominated by individuals of one genetic cluster and different riverine collection sites were dominated by individuals of another genetic cluster. The phylogenetic analysis revealed that all the riverine populations of Hilsa shad were further subdivided into the north-western riverine (turbid freshwater) and the north-eastern riverine (clear freshwater) ecotypes. Among all of the putatively adaptive loci, only 36 loci were observed to be in the coding region, and the encoded genes might be associated with important biological functions related to the local adaptation of Hilsa shad. In summary, our study provides both neutral and adaptive contexts for the observed genetic divergence of Hilsa shad and, consequently, resolves the previous inconclusive findings on their population genetic structure across their diverse migratory habitats. Moreover, the study has clearly demonstrated that NextRAD sequencing is an innovative approach to explore how dispersal and local adaptation can shape genetic divergence of non-model anadromous fish that intersect diverse migratory habitats during their life-history stages.

## 1. Introduction

The Hilsa shad *Tenualosa ilisha* (subfamily Alosinae) is an anadromous and important trans-boundary fish that lives in the Bay of Bengal and migrates to the upstream rivers of Bangladesh (86% share), India (8% share), and Myanmar (4% share) for feeding, breeding, and the nursing of offspring [1,2]. Since 2016, the annual production of Hilsa shad has increased by more than 0.5 million tons, and the species alone has been contributing approximately 44% to the captured fish production in Bangladesh, which accounts for 10.5% of the total annual fish production, representing approximately 1% of the total gross domestic product of the country [3]. While the production of Hilsa shad has increased in recent years, the majority of the increased production comes from the sea and estuaries [3]. However, the production of the upstream migratory rivers remained stable or even decreased due to the disruption of migratory routes by heavy siltation, loss of spawning, feeding, and nursing grounds, the indiscriminate catching of juveniles, and an increased fishing of adults [1]. Moreover, migrations to the upstream rivers during the breeding season are largely influenced by the climatic conditions, particularly the southwest monsoon, which results in the flooding of the major rivers in Bangladesh, India, and Myanmar. All of these natural and human-induced activities together put the upstream Hilsa shad fishery at high risk [2]. As an anadromous fish, Hilsa shad migrates in heterogenic environmental conditions across complex aquatic systems, which primarily differ in terms of salinity, turbidity, and other ecological factors. Fishes fundamentally acclimate to the changing environmental conditions by expressing particular adaptive phenotypes, as short-term responses, while eventually adapting to the prevailing environmental conditions by changing their genetic components in response to the new local selective pressure [4]. Therefore, it is the major concern for the fishery managers and scientists along the Bay of Bengal regions to understand and predict how the Hilsa shad populations respond to the imposing selective pressure that results in a genetic variation among populations.

While Hilsa shad is largely anadromous, it has also been indicated that the fish may follow an amphidromous migratory pattern, because both immature and mature fish often migrate between marine and freshwaters not only for breeding, but also for feeding purposes [2]. In addition to the anadromous type, recent studies have also reported two other ecotypes—a fluvial potamodromous type and a marine type, along the Bay of Bengal regions [5,6,7]. The potamodromous stocks remain in the river systems and complete their life cycle within freshwater habitats, while the marine ecotype inhabits nearshore coastal and sea habitats and relies on downstream estuarine waters for spawning [5,6,7]. Therefore, understanding the degree of genetic erraticism among the different ecotypes is crucial for the sustainable exploitation, conservation, and management of the Hilsa shad population. However, the genetic structure and the divergence patterns of the Hilsa shad populations among the different migratory ecotypes have remained obscure until today.

The discrimination of populations, as management units, needs to be overtly implemented for the sustainable exploitation of the anadromous fish species. However, most of the anadromous fish species are distributed across diverse habitat types, causing natural selection to play an important role in promoting genetic discrimination and local adaptation [8]. As an anadromous fish, Hilsa shad populations also have a very weak genetic discrimination across their migratory habitats due to their high rates of gene flow, highly connected divergent population, and large effective population size [8,9,10]. Moreover, population boundaries are often elusive in Hilsa shad, and a lack of obvious stock boundaries often confounds the discrimination of spatial management units and the degree of connectivity among different populations [11,12]. Therefore, it is necessary to investigate the genetic structure of Hilsa shad by a combined analysis of both neutral and adaptive loci [10,13]. However, only neutral genetic markers have been extensively used in the Hilsa shad in recent decades. The limited number of neutral markers used, and their insufficient power to discriminate fine-scale genetic structures due to the recent divergence in their large population size, has caused the results concerning the structuring pattern of Hilsa shad populations to be inconclusive [14,15,16,17,18,19]. Moreover, assessing the fish population structure by the simultaneous use of both neutral and adaptive markers is yet to be common along the Bay of Bengal regions [20,21]. 

The recent development of next-generation sequencing (NGS) techniques has expanded the prospects of providing insights into the molecular basis of local adaptation for fish with a low genetic differentiation. This technique allows for the genotyping of thousands of genome-wide single nucleotide polymorphism (SNP) loci for ecologically important fish, without any reference genomes, to discover a large number of genetic markers [22]. The discovery of a large number of genetic markers not only affords a higher statistical power in discriminating the populations, but also facilitates the exploration of the putative non-neutral signatures regulating the underlying adaptive traits [23,24], induced either by natural [25] or anthropogenic factors [26,27]. Moreover, they provide a better solution, where traditional neutral markers fail, for distinguishing genetically weak structured populations with clear population boundaries [28,29,30,31,32]. At present, restriction site-associated DNA sequencing (RAD-seq) is widely used to genotype thousands of SNPs in multiple individuals of anadromous and other marine fish species at a relatively low cost [33,34]. Moreover, the NextRAD method has also recently gained popularity in overcoming the restriction fragment length bias of RAD-seq using Nextera library preparation and selective PCR primers to consistently amplify genome-wide loci between samples [35]. Various outlier tests have proven to be a popular means for the discovery of the putatively adaptive markers of highly dispersive anadromous fish species [8,12]. As Hilsa shad have a very weak genetic discrimination across their heterogenous migratory habitats, it represents an ideal candidate for identifying a putatively adaptive panel of SNP loci, generated by a genome scan using the NextRAD method and outlier tests.

The degree of genetic discrimination among the Hilsa shad populations, available in different migratory routes, has remained obscure until today. Therefore, the aims of the present study are to discriminate the fine-scale genetic divergence of Hilsa shad populations and to reconcile previous inconclusive findings on the population genetic structure across their diverse migratory habitats. In this study, we employed the NextRAD sequencing technique to genotype 15,453 SNP loci for 300 individuals of Hilsa shad, collected from nine strategic habitats, including sea, estuary, and different rivers in Bangladesh. The different spatial analyses, using the entire 15,453 SNP dataset, revealed a very weak genetic discrimination among the different population of Hilsa shad. Therefore, we applied both the F_ST_ OutFLANK and pcadapt approaches to ascertain a set of neutral and adaptive genetic markers to better understand whether there is a neutral and/or adaptive genetic variation associated with their distribution in heterogenic environments. Finally, we conducted a homology search to align the NextRAD sequence tags of the adaptive loci to the known genes in public databases in order to elucidate the homologous functions of these genes, which might be associated with the divergence local adaptation of Hilsa shad.

## 2. Materials and Methods

### 2.1. Habitats of T. ilisha in the Complex Ecosystem of Bangladesh

**Figure 1:**
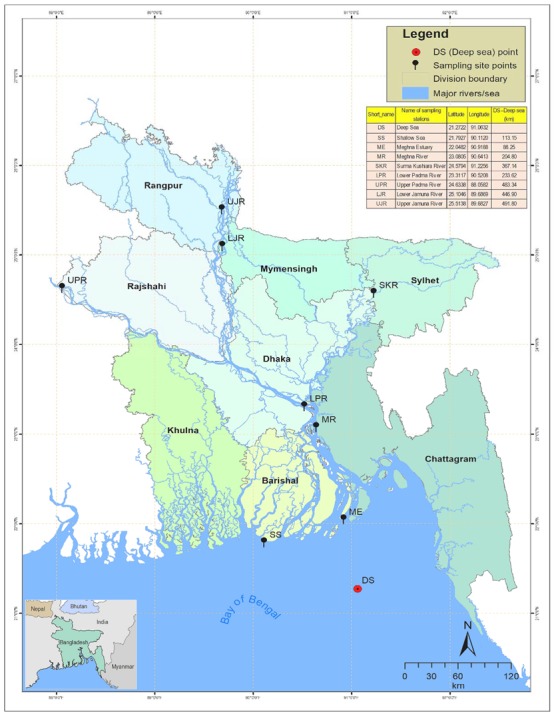
Map of the anadromous Hilsa shad *Tenualosa ilisha* collections sites across its diverse migratory habitats, including deep sea, shallow sea, estuaries, and different rivers in Bangladesh. DS, deep sea; SS, shallow sea; ME, Meghna estuary; MR, Meghna river; SKR, Surma–Kushiara river; LPR, lower Padma river; UPR, upper Padma river; LJR, lower Jamuna river; UJR, upper Jamuna river.

In the geographical region of Bangladesh, Hilsa shad mainly inhabits the Bay of Bengal (sea), including the lower regions of the estuary (Meghna estuary), but it migrates to the upstream rivers, mainly the Ganges–Brahmaputra–Meghna (GBM) river systems, during the spawning season and returns to its original habitat, after spawning, in adulthood. Jamuna River (also known as the Brahmaputra) joins the Padma River (also known as the Ganges) in Goaland, in the Rajbari district of Bangladesh, and the combined flow of the Padma and Jamuna Rivers flow downwards, forming the Padma River. The Meghna, coming from the northeast region of the country, joins with the Padma further downstream in Chandpur and flows downwards to the south, forming the Meghna River, which finally meets the Bay of Bengal through the formation of a large estuary, named the Meghna Estuary (Figure 1). 

According to the geography of Bangladesh, the Padma–Jamuna Rivers are considered to be the northwestern rivers, which together discharge about 1 billion tons of sediment annually, placing the Padma–Jamuna in the top three rivers in the world [36]. Therefore, the western rivers are considered to be turbid rivers, with suspended sediment concentrations of about 190–1600 mg/l in the Padma and 220–1400 mg/l in the Jamuna [37]. The Meghna River is formed inside Bangladesh, in the Kishoreganj District, by the joining of the Surma and the Kushiyara Rivers, both of which originate in the hilly regions of Eastern India. Various tributaries of the Surma and Kushiyara flow through a vast natural depression, called the *haor* basin (*haor* is a wetland ecosystem of 80,000 sq·km in Northeastern Bangladesh, which is surrounded by the hill ranges of India), where waters settle the sediment loads, before a confluence, named the Meghna. Due to these characteristics, the Meghna River and its tributaries are considered to be clear water rivers, and their suspended sediment concentrations are about 20–750 mg/l [37], but the higher range is only observed occasionally during periods of heavy rainfall. The salinity varies from 30.3–33.4 ppt in the Bay of Bengal, 2.5 to 25.6 ppt in the Meghna Estuary, and <1.0 ppt in the different freshwater river systems of Bangladesh [38]. 

### 2.2. Sample Collection of T. ilisha

Three hundred individuals of *T. ilisha* were collected from nine strategic habitats across the migratory routes of Hilsa shad, including sea, estuary, and major freshwater river systems in Bangladesh (Figure 1, Table 1). For the present study, 30 individuals were collected from each sampling site, while 60 individuals were collected from the upper Padma River (UPR). Fin clips of Hilsa shad samples were preserved in absolute ethanol, prior to DNA extraction. DNA isolations were performed using the Promega DNA purification system (Promega, Madison, WI, USA), according to the manufacturers’ protocol. DNA quantifications were conducted using the real-time PCR fluorescence measurements of double stranded DNA and the Quant-it kit (Life Technologies, Foster City, CA). All samples were collected under a Bangladesh government permit and in accordance with the animal care protocol (CVASU20180828), as approved by the Chattagram Veterinary and Animal Sciences University’s Animal Care and Biosafety Committee. 

### 2.3. NextRAD Genotyping

In this method, NextRAD genotyping-by-sequencing libraries were prepared at SNPsaurus (SNPsaurus, LLC, Eugene, OR, USA) by fragmenting the genomic DNA [39,40] with the Nextera DNA Flex Library Prep Kit (llumina, San Diego, CA, US), which also ligated short adapter sequences to the ends of the fragments. To adjust the quality of the DNA and increase the fragment size, the Nextera reaction was scaled at 10 ng of genomic DNA, as an input in the fragmentation reaction, although 20 ng of genomic DNA was also used to compensate for the amount of degraded DNA in the samples. The fragmented DNA was then amplified for 25 cycles at 72 °C, with one of the primers matching the adapter and extending 8 nucleotides into the genomic DNA, with the selective sequence, GTGTAGAG. Thus, only fragments starting with a sequence that could be hybridized by the selective sequence of the primer were efficiently amplified. The NextRAD libraries were sequenced on an Illumina HiSeq 4000, with eight lanes of 150 bp reads (University of Oregon, Eugene, OR, USA). The resulting fragments were fixed at the selective end and had different lengths, depending on the initial Nextera fragmentation. Therefore, a careful size selection of the library was not required, as the amplified DNA from a particular locus was present at many different sizes.

### 2.4. Sequence Assembly, Filtering and Discovery of SNPs

The reads were trimmed using bbduk in custom scripts of SNPsaurus, LLC (BBMap tools, Eugene, OR, USA) http://sourceforge.net/projects/bbmap/): bbmap/bbduk.shin=reads/run_ 1612/1612_GTAGAGGACTAAGCCT_S483_L006_R1_001_subset.fastq.gzout=reads/run_1612/1612_GTAGAGGACTAAGCCT_S483_L006_R1_001_t.fastq.gz ktrim=r k=17 hdist=1 mink=8 ref=bbmap/resources/nextera.fa.gz minlen=100 ow=t qtrim=r trimq=10. A de novo reference was created by evenly collecting a total of 10 million reads from the samples and excluding reads that had counts of <7 or >1000. The remaining loci were then aligned to each other to identify allelic loci and collapse allelic haplotypes into a single representative. All reads were mapped to the reference, with an alignment identity threshold of 0.95, using bbmap (BBMap tools). Genotype calling was conducted using callvariants (BBMap tools) (callvariants.shlist=ref_shadupsplit_rm.txt.align_samplesout =shadupsplit_total.vcfref=h_GCA_004329175.1_ BAU_Tilisha_2.1_genomic.fna ploidy=2 multisample=t rarity=0.05 minallelefraction=0.05 usebias=f ow=t nopassdot=f minedistmax=5 minedist=5 minavgmapq=15 minreadmapq=15 minstrandratio=0.0 strandedcov=t). The vcf was filtered to remove alleles with a population frequency of <3%. Loci that were heterozygous in all samples or had more than 2 alleles in a sample (suggesting collapsed paralogs) were removed. The absence of artifacts was checked by counting the SNPs at each read nucleotide position and determining that the SNP number did not increase with the reduction of the base quality at the end of the read. 

The original shadstringent.vcf file contained data for 26,718 SNPs loci, existing within a catalog of 92,721 consensus NextRAD tagged sequences of 150 bases each. A column containing a unique ID for each SNP locus was added to the unfiltered vcf file, using a custom perl script to remove the redundancy among loci. Further filtering steps included the removal of complex SNPs with more than two alleles, an overall minor allele frequency of less than 5%, and a completeness of data among samples of less than 80%. Likewise, samples containing a completeness of data of less than 80% among the remaining loci, a minimum quality score lower than 30, and a mean depth per genotype lower than 20 were removed from the dataset. Additionally, where there were multiple SNPs located in a single NextRAD sequence tag, only the first cataloged SNP was kept in the dataset so as to avoid the possibility of a single NextRAD locus having a disproportionate effect on the analyses. The unique IDs for the SNP loci that passed quality control standards were transferred to a whitelist, which was used to filter the original vcf file using a custom perl script. After all of the filtering steps were complete, a total of 15,453 individual SNP loci remained in the dataset.

### 2.5. Clustering Analyses

The initial clustering analyses, through several spatial structuring programs using all 15,453 putative SNPs loci, revealed extremely low to no genetic structuring among all collected individuals from different environments. Therefore, we identified outlier SNPs using the adegenet v2.0.1 R package [41] and two different approaches, OutFLANK [42] and pcadapt version 3.0.2 [43,44]. OUTFLANK calculates the neutral distribution of Fst values and then uses this distribution to assign q-values to each locus to detect adaptive loci that are putatively influenced by selection [42]. On the other hand, pcadapt performs a principal component analysis and computes the *p*-values of each locus to detect adaptive loci [43,44]. Default parameters were used for OUTFLANK and pcadapt analysis, and the “number_of_samples” parameter was set to 9 (a number equal to the sampled collections). To control the false positive, we set the F_ST_ threshold value to 0.05 in the OUTFLANK approach and the false discovery rate (FDR) threshold value to 0.05 in the pcadapt approach. A neutral loci dataset and an adaptive loci dataset were created from the output of these two approaches for all downstream analyses. The GenoDive version 3.0 (Universiteit van Amsterdam, Amsterdam, The Netherlands) was used to conduct analysis of molecular variance (AMOVA) analyses on both datasets [45]. The GenoDive was also used for significance testing of pairwise F_ST_ to determine the genetic differences between collection sites for the neutral and outlier datasets using the default settings, with the samples grouped by collection site. An isolation by distance analysis, using the outlier dataset, was conducted in the “adegenet” R package, using a pairwise distance matrix for all collection sites. Significance testing for the isolation by distance analysis was conducted through a Mantel test, using an Edward’s genetic distance matrix and a physical distance matrix between the collection sites, with 9999 iterations (also using the “adegenet” package). In addition, each adaptive locus was subjected to a BLASTn search of all sequences in the NCBI non-redundant database (word size = 11; mismatch scores = 2 and −3; and maximum e-value = 1 × 10^−5^). Principal component analysis (PCA) was conducted using the adegenet R package (Universite´ de Lyon, UMR 5558, France) and both the neutral and outlier datasets. The Bayesian clustering method, implemented in the STRUCTURE software v. 2.3.4 (Standford University, Stanford, CA, USA), was used to genetically assign individuals to clusters [46]. Simulations were run for 100,000 steps, following a burn-in period of 100,000 steps, considering values of K (number of clusters) from one to 15, with 10 replications for each value of K. The analysis was performed using an admixture, correlated allele frequencies, and no prior information on the sampling location or morphological species. For each individual, the program identifies the fraction of the genome that belongs to each one of the clusters. The rate of change in the log likelihood between successive K values was also estimated [47]. The calculations were performed using STRUCTURE HARVESTER [48]. The clusters of the estimated population structure were visualized using CLUMPAK [49]. Clustering analysis, implemented with the discriminate analysis of principal components (DAPCs) and the outlier dataset, was also conducted, using “adegenet” to reveal possible genetic clustering among samples, without grouping by collection site.

### 2.6. Data Accessibility

Our raw data for 300 individuals were submitted to the DDBJ (https://ddbj.nig.ac.jp), with the DRA accession number: DRA009185. The data is publicly available from 24 December 2019. 

## 3. Results

### 3.1. NextRAD Sequencing and Outlier Analysis

In this study, we sequenced 100,000 loci at a depth of 20× using the NextRAD-genotyping technique, implemented on an Illumina HiSeq 4000 platform, for each individual of 300 *T. ilisha* fish, which, on average, gave two million reads of 150 bp per individual. The sequence analysis revealed that all of the 300 individual fish had a completeness of the genotyping data of more than 80%; therefore, all of the *T. ilisha* samples were considered for further downstream analyses. Out of total 46,307 SNP loci, within a catalog of 94,738 consensus sequences, only 26,718 SNP loci remained, after the indels and SNP sites with a minor allele frequency of less than 5% had been removed from the original dataset. Further quality filtering by variant calling analysis resulted in 15,453 putative SNPs loci from all 300 individuals, and these were finally used for the fine-scale population genetic structuring of *T. ilisha*.

**Figure 2:**
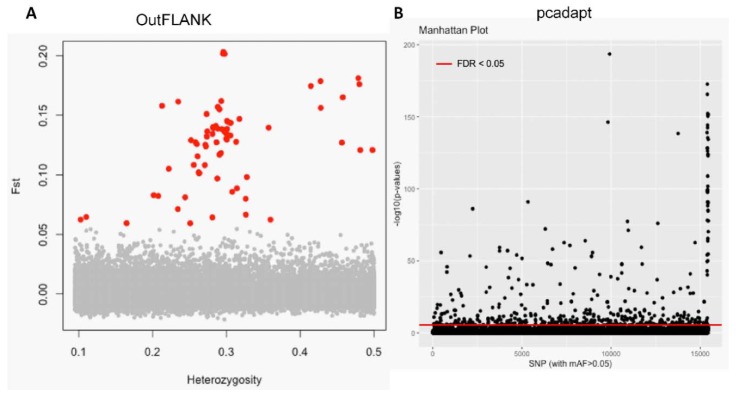
Separation of the putatively adaptive and neutral panels of single nucleotide polymorphisms (SNPs) loci, based on the F_ST_ OutFLANK and pcadapt approaches. Among the 15,453 SNP loci, the F_ST_ OutFLANK approach identified 74 SNPs (red circles) (**A**), whereas the pcadapt approach detected 449 SNPs, as putative adaptive loci (above the red line) (**B**). The remaining were considered to be putatively neutral loci.

**Figure 3:**
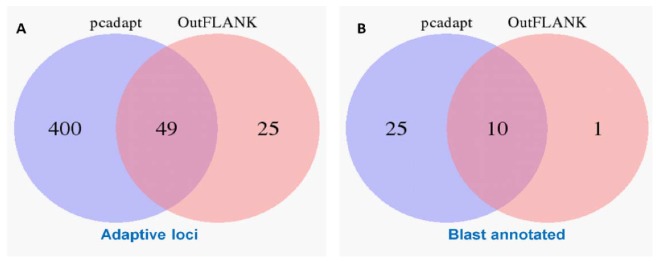
Venn diagram showing the similarity of the putatively adaptive panel of SNP loci (**A**) and a blast annotation of these loci in the coding regions of the known genes (**B**) using the F_ST_ OutFLANK and pcadapt approaches.

The initial clustering analyses using the 15,453 putative SNPs loci revealed very little genetic differentiation among different collection sites. Therefore, we applied both the F_ST_ OutFLANK and pcadapt approaches to identify the putative adaptive loci of *T. ilisha* under local selection pressure. Out of the 15,453 polymorphic SNPs loci, 74 SNPs loci were identified as outliers and putatively under positive selection by F_ST_ OutFLANK, while 449 SNPs loci were detected as candidate outlier loci by the pcadapt approach (Figure 2). Among this putative panel of SNPs loci, 49 loci were identified as being common between the two approaches (Figure 3). The remaining 15,379 and 15,004 SNPs were considered as putatively neutral loci by the F_ST_ OutFLANK and pcadapt approaches, respectively.

### 3.2. Demographic Inferences from F_ST_ Statistics and AMOVA Analysis

A pairwise F_ST_ comparison showed distinct values when only the outlier or neutral SNPs were used (Table 2). An apparent difference was also observed between the pairwise F_ST_ comparisons for the outlier SNPs, identified by both the F_ST_ OutFLANK and pcadapt approaches. F_ST_ estimation, based on putatively neutral SNPs, was low but significant, ranging between 0.001 and 0.004 (*p* ≤ 0.001). As expected, the outlier SNPs detected by the F_ST_ OutFLANK and pcadapt approaches showed higher significant F_ST_ values than the neutral SNPs, with a range of 0.020 to 0.327 (*p* ≤ 0.001) and 0.005 to 0.073 (*p* ≤ 0.001), respectively. In both approaches, higher F_ST_ values were observed between the Meghna estuary (ME) and upper Jamuna river (UJR) (0.327, *p* ≤ 0.001; 0.073, *p* ≤ 0.001) and deep sea (DS) and UJR (0.315, *p* ≤ 0.001; 0.070, *p* ≤ 0.001) collection sites. As anticipated, non-significant and lower F_ST_ values were observed between the collection sites from the two locations of sea, the sea and estuary, and the lower and upper portion of the same rivers and/or their tributaries.

For the putative adaptive panel of SNP loci, the AMOVA analysis yielded overall F_ST_ values of 0.097 and 0.015 for the F_ST_ OutFLANK and pcadapt approaches (*p* < 0.001), respectively (Table 3). Interestingly, hierarchical AMOVA, based on the adaptive loci identified by the F_ST_ OutFLANK and pcadapt approaches, revealed a high divergence between individuals (103.1% and 101.7%) but no differentiation among individuals (−12.8% and −3.2%). A substantially low but significant divergence (*p* < 0.001) was observed (9.7% and 1.5%) among the different collection sites of Hilsa shad. Similarly, an overall F_ST_ value of 0.002 was observed in the putative panel of neutral SNP loci identified by both approaches (*p* < 0.001). Consistent with the putatively adaptive loci, the AMOVA analysis of the neutral loci for both approaches demonstrated a 100.5% variation between individuals, no variation (−0.7%) among individuals, and a substantially low (0.2%) but significant variation (*p* < 0.001) among the different collection sites of Hilsa shad (Table 3).

### 3.3. Genetic Structure and Recruitment Assignment Based on Different Spatial Analyses

**Figure 4:**
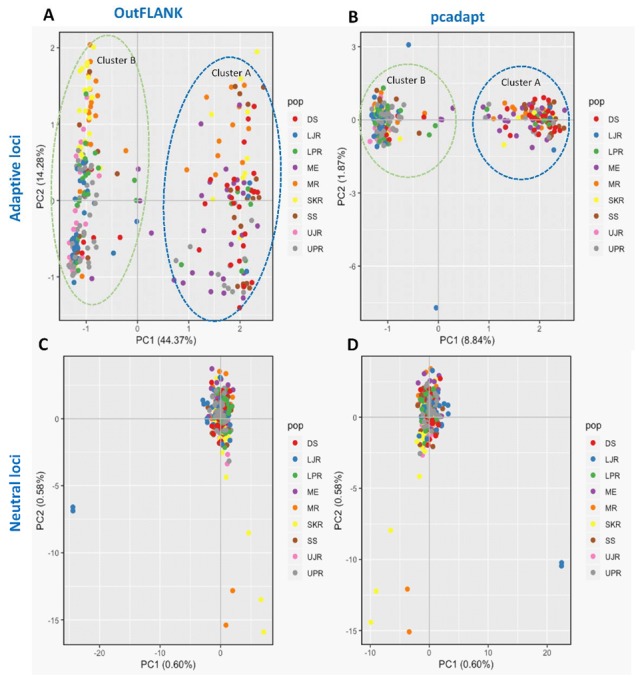
Scatterplots showing the principal components analysis (PCA) for: (**A**,**B**) The putatively adaptive panels of SNPs in the F_ST_ OutFLANK (**A**) and pcadapt approaches (**B**); and (**C**,**D**) the putatively neutral panel of SNP loci in the F_ST_ OutFLANK (**C**) and pcadapt approaches (**D**). DS, deep sea; SS, shallow sea; ME, Meghna estuary; MR, Meghna river; SKR, Surma–Kushiara river; LPR, lower Padma river; UPR, upper Padma river; LJR, lower Jamuna river; UJR, upper Jamuna river.

We have also observed substantial differences in genetic structure patterns, inferred from several spatial analyses based on the outlier and neutral SNPs datasets. The principal components analysis (PCA) of the outlier SNPs, using both the F_ST_ OutFLANK and pcadapt approaches, showed two well-separated clusters, A and B. Sea and estuarine (salty and brackish water) collection sites were dominated by individuals of cluster A, while different riverine (freshwater) collection sites were dominated by individuals of Hilsa shad of cluster B (Figure 4). However, no clustering was found from the PCA analysis of the neutral panel of the SNP datasets. 

**Figure 5:**
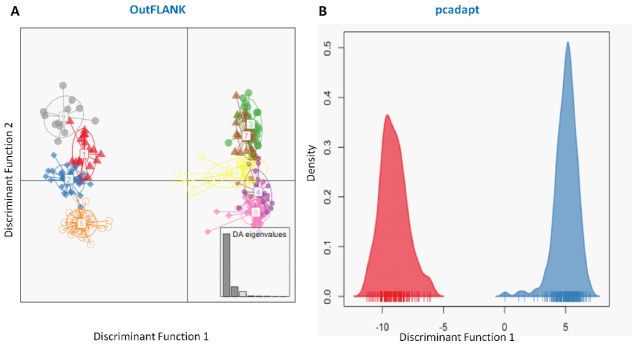
Plots showing the discriminant analysis of principal components (DAPCs) of genetic differentiation for the putatively adaptive panels of SNP loci in the F_ST_ OutFLANK (**A**) and pcadapt approaches (**B**). The DAPCs of the putatively adaptive panels of SNP loci in the F_ST_ OutFLANK (**A**) and pcadapt (**B**) approaches consistently determined two major clusters. One group of clusters was mostly dominated by the marine–estuarine population, and the other group was dominated by the riverine population.

**Figure 6:**
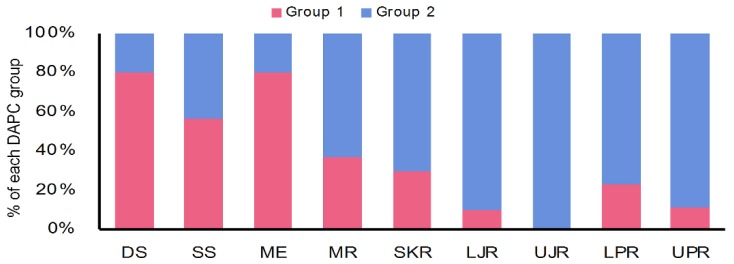
DAPCs grouping of *T. ilisha* individuals for the putatively adaptive SNPs identified by the pcadapt approach based on the percentages of the each DAPCs clusters according to their different collection sites. The DAPCs grouping of the putatively adaptive panels of SNP loci identified by the pcadapt approach of different Hilsa shad collection sites further confirmed that the marine–estuarine collection sites (DS, SS, and ME) are mostly dominated by individuals of Group 1, whereas all the riverine sites (MR, SKR, LJR, UJR, LPR, and UPR) are mostly dominated by individuals of Group 2. DS, deep sea; SS, shallow sea; ME, Meghna estuary; MR, Meghna river; SKR, Surma–Kushiara river; LPR, lower Padma river; UPR, upper Padma river; LJR, lower Jamuna river; UJR, upper Jamuna river.

Similarly, the discriminant analysis of principal components (DAPCs) scatterplot on the putatively adaptive SNPs identified by the pcadapt approach also revealed two genetic clusters (Figure 5B). However, DAPCs analysis for the putatively adaptive SNPs identified by the F_ST_ OutFLANK approach demonstrated nine clusters at a finer-scale level under two major clusters, as revealed for the outlier datasets identified by the pcadapt approach (Figure 5A). Furthermore, individual assignment data of Hilsa shad by DAPCs analysis for the putatively adaptive SNPs identified by the pcadapt approach were presented in percentages of the two DAPCs clusters according to their different collection sites (Figure 6). Mixed ancestry patterns of the Hilsa shad individuals were detected in the DAPCs grouping of each collection site, except for the upper Jamuna river (UJR). Consistent with the previous results, DAPCs grouping also demonstrated that marine and brackish water collection sites (DS, SS, and ME) were mostly dominated by the individuals of Group 1, while different riverine collection sites (MR, SKR, LJR, LPR, and UPR) were mostly dominated by the individuals of the Group 2 (Figure 6).

**Figure 7:**
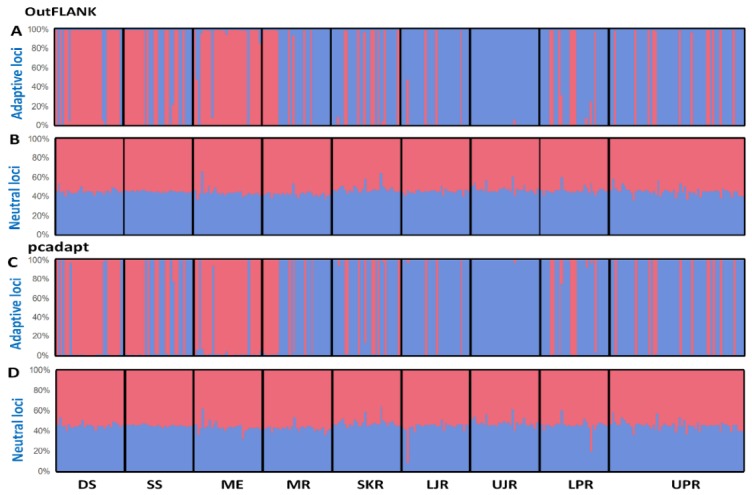
Bayesian STRUCTURE bar plot, based on the (**A**,**B**) F_ST_ OutFLANK approach for the 74 putatively adaptive SNP loci (**A**) and 15,379 putatively neutral SNP loci (**B**); and (**C**,**D**) pcadapt approach for the 449 putatively adaptive panel of SNP loci (**C**) and 15,004 putatively neutral panel of SNP loci (**D**). Black lines separate the individuals of different populations. Each vertical line represents an individual. The colors represent the proportion of inferred ancestry from the K ancestral populations. Based on the delta K statistic, the best-supported number of a posteriori genetic clusters was K = 2 for the standard admixture model. DS, deep sea; SS, shallow sea; ME, Meghna estuary; MR, Meghna river; SKR, Surma–Kushiara river; LPR, lower Padma river; UPR, upper Padma river; LJR, lower Jamuna river; UJR, upper Jamuna river.

The multivariate methods were in complete concordance with the results obtained using the Bayesian clustering approach, implemented in STRUCTURE. Our STRUCTURE results, with an optimal value of K = 2, were supported by highest log-likelihood values, where K = 2, which was followed by their exponential decline as the number of groups increased. No evidence of a population structure was shown in the neutral loci datasets, as the genetically homogenous group may suggest that *T. ilisha* is composed of a single panmictic population (Figure 7B,D). In contrast, a clear population subdivision, with a very small degree of admixture, was observed in the outlier datasets (Figure 7A,C). 

**Figure 8:**
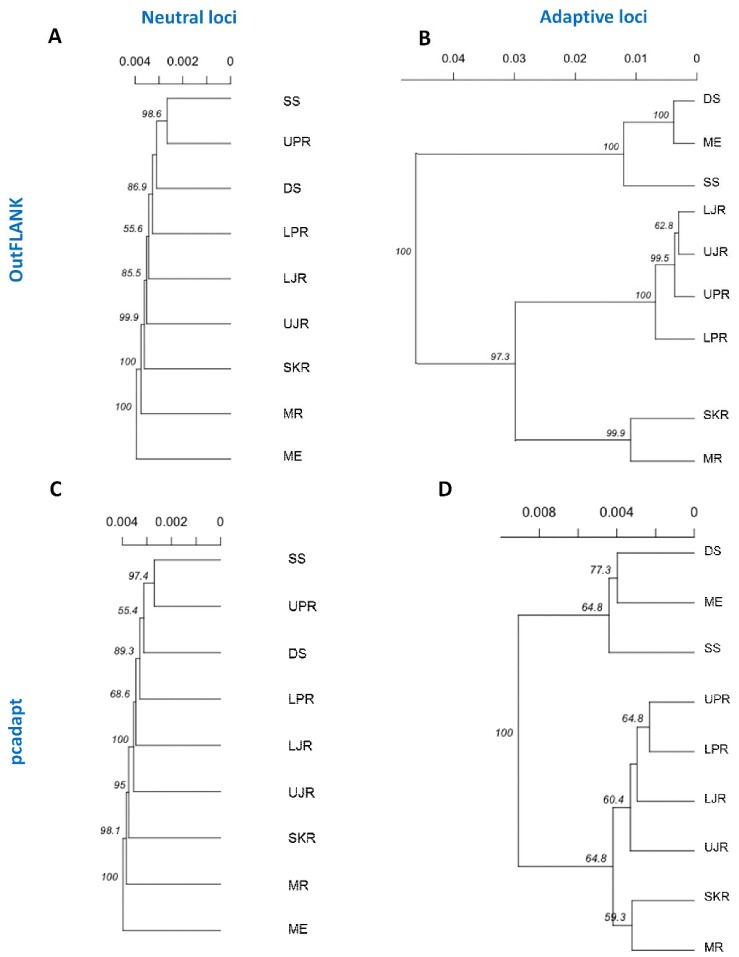
Neighbor-joining trees, based on the Nei’s genetic distances using the following sets of loci: (**A**,**B**) The putatively neutral panel of SNP loci in the F_ST_ OutFLANK (**A**) and pcadapt approaches (**B**); and (**C**,**D**) the putatively adaptive panel of SNP loci in the F_ST_ OutFLANK (**C**) and pcadapt approaches (**D**). Branch nodes are denoted as the percentage of bootstrap support, which was generated with 1000 replicates. DS, deep sea; SS, shallow sea; ME, Meghna estuary; MR, Meghna river; SKR, Surma–Kushiara river; LPR, lower Padma river; UPR, upper Padma river; LJR, lower Jamuna river; UJR, upper Jamuna river.

These clustering patterns were further confirmed by neighbor-joining (NJ) trees, reconstructed based on Nei’s genetic distances (Figure 8). The NJ trees results consistently revealed that a divergent local adaptation in different migratory habitats divided Hilsa shad population into two genetically structured ecotypes: (1) Marine and estuarine, and (2) riverine groups. Interestingly, the riverine *T. ilisha* collection sites were further separated into two sub-clusters: The north-western riverine (turbid freshwater) and the north-eastern riverine (clear freshwater) ecotypes.

**Figure 9:**
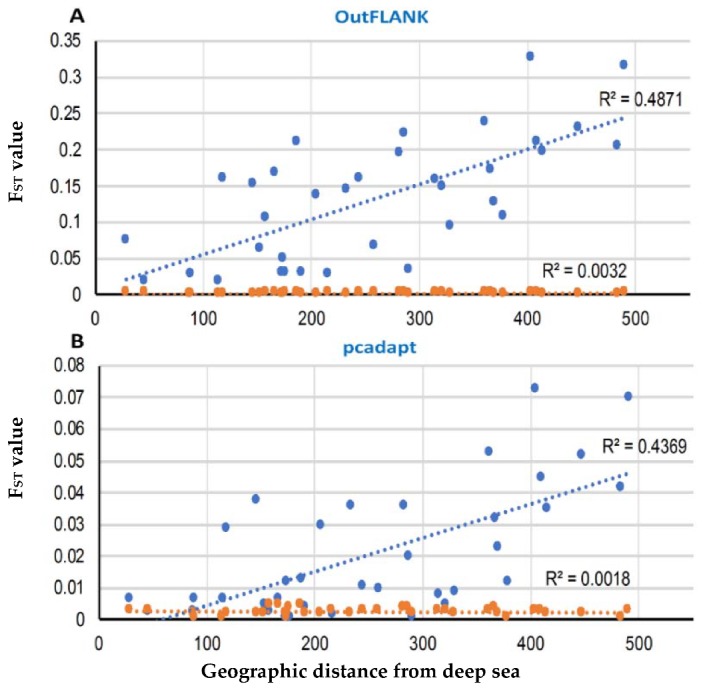
Correlation of the pairwise F_ST_ value with the pairwise geographical distances among the *T. ilisha* collection sites in the F_ST_ OutFLANK (**A**) and pcadapt approaches (**B**). The blue circle represents the putatively adaptive loci F_ST_, and the red circles are the putatively neutral loci F_ST_. The Mantel test showed highly significant effects based on 9999 replicates.

A Mantel test, based on the outlier loci datasets, identified by both F_ST_ OutFLANK and pcadapt approaches, revealed a strong and significant relationship between the genetic and geographic distance, with R^2^ = 0.4871 (*p* < 0.01) and R^2^ = 0.4369 (*p* < 0.01), respectively. Analyses of the neutral dataset indicated a high genetic connectivity and weak differentiation between the populations by geographical distance (Figure 9).

### 3.4. Gene Function of the Outlier Loci

The BLAST search of the SNP flanking sequences showed that 35 out of 449 (7.8%) outlier loci identified by the pcadapt approach and 11 out of 74 (13.9%) outlier loci identified by the F_ST_ OutFLANK approach were observed to be in the protein coding region, and their gene functions are depicted in Table 4. Among these putatively adaptive protein coding loci, five loci (SCED01086260.1:268, SCED01010228.1:165, SCED01066042.1:124, SCED01005892.1:34793, and SCED01010418.1:19123) that encode the genes (nrxn3b, TRIM67, Grik2, GABBR1, and Herc1) are mostly involved in the different neuronal activity, and important for neural communication and responsible to control a range of behavioral phenotypes (Table 4). The majority of the outlier loci (SCED01072826.1:68, SCED01077141.1:127, SCED01094462.1:170, SCED01001444.1:144867, SCED01011615.1:5090, SCED01010819.1:27239, SCED01000975.1:25910, SCED01000852.1:137973) were found to encodes genes which are mostly responsible for the metabolic process, particularly in glucose, glycogen, and lipid metabolism. Another two outlier loci (SCED01000696.1:30199 and SCED01008207.1:36816) are associated with two genes, namely, KDM6A and UBR3, which play a direct role in growth and embryonic development processes. Other loci encode the genes mainly involved in different biological processes, such as cell regulation and biosynthesis, membrane transport, mitotic cell development, metabolic process, signal transmission, and other functions (Table 4). The unknown function of other outlier loci did not meet the significant alignment with publicly available sequences.

## 4. Discussion

Revealing the spatial and temporal dispersal of fish stocks is important for the systematic monitoring and management of fish populations. This study applied NextRAD-based data to assign Hilsa shad individuals to their respective origin populations to explore the variation in stock components and catch compositions. Loci with a high resolving power and potentially important local adaptation to the environment were detected and validated. We observed a remarkable differentiation between the individuals of marine and estuarine collection sites in the south and freshwater riverine collection sites in the north of Bangladesh, with a slight mixing between them, confirming the general validity of the stock designations. In contrast to traditional markers for detecting genetic differentiation, the genomic approach identifies the traces of selection that shape population divergence [50]. Genetic differentiation, under selective force, is not unprecedented, given that analysis based on markers under selection provides more structuring, compared to neutral markers [29,51]. This type of analysis is more promising for populations with a large effective size and minimal genetic drift effect [52], where a high level of gene flow may not be efficient to dilute the differentiation effects driven by adaptive markers [30]. The higher level of assignment success of NextRAD sequencing is likely due to larger number of loci and also outlier loci with a high discriminatory power. 

We observed a contrasting magnitude and contrasting patterns of genetic variation in both neutral and outlier loci across multivariate and individual-based analyses (PCA, DAPCs, STRUCTURE, AMOVA, and NJ phylogenetic). The neutral set of markers showed an extremely low to no genetic structuring between all collection sites individuals from the different environments. Pairwise F_ST_ estimates, based on the neutral dataset, indicates that a large fraction of the genome is undifferentiated among the collection sites, with 19.4% of estimates being <0.001 (*p* ≤ 0.001) (Table 2). As expected, it is not unpredicted to observe a panmictic structure in all the other analyses, when the entire dataset is considered. In contrast, our analyses based on the outlier dataset provided evidence of a significant genetic differentiation between the populations of the freshwater and marine environments. Our isolation by distance (IBD) results also suggested that the genetic structure demonstrates the effect of dispersion and selection for the outlier dataset, and these factors should be considered when delineating conservation and management units [50]. The outlier loci suggest adaptive responses to potential environmental factors, such as salinity, which vary on regional scales, while neutral loci are influenced by genetic drift [52]. Given that the divergences revealed by the outlier dataset are discordant with the neutral loci, these loci should be driven by the effects of selection and not by the isolation alone [53]. 

The lack of genetic structure in the neutral loci indicates that all Hilsa shad individuals from different collection sites are connected via high levels of gene flow between the marine water and freshwater environments, which is consistent with previous studies using allozyme [14,15], restriction fragment length polymorphism (RFLP) [16,17], random amplification of polymorphic DNA (RAPD) markers [54,55,56,57], and mitochondrial DNA cytochrome *b* gene nucleotide sequencing [18,19]. The genetic divergence depicted by the outlier loci dataset suggests that dispersion may likely happen in the foraging ground in marine and brackish water habitats, allowing for a substantial gene flow among distant locations. The shallow sea (SS), deep sea (DS) and Meghna estuary (ME) populations are genetically homogenized, based on clustering patterns in multivariate (PCA, DAPCs) and individual analyses (STRUCTURE, NJ phylogenetics). It is also possible that physical barriers between the different environments are semipermeable, allowing for constraint dispersal, while the environmental contrasts between these habitats reinforce those barriers through selection [53]. The expansions of founder populations may also occur after isolation, which further induces adaptation, if colonizing individuals carry beneficial mutated genes [58]. 

Our results concerning the outlier loci also indicate that the local conditions are sufficiently different to prevent the genetic homogenization of the Hilsa shad individuals collected from the different geographical regions through the selection process [10]. We observed that the Hilsa shad from the deep sea (DS) and upper Jamuna river (UJR) collection sites in the outlier loci dataset, as detected by F_ST_ OutFLANK (F_ST_ = 0.315, *p* ≤ 0.001), was an order magnitude greater than those in the putatively neutral loci dataset (F_ST_ = 0.003, *p* ≤ 0.001). While it is difficult to distinguish between differentiations caused by selection or drift processes [53], it is noteworthy that these processes are mutually inclusive and likely act in concert on populations found around the region, given its complex anadromous life cycle. The presence of two major clusters of Hilsa shad in Bangladesh was substantiated by these analyses of the outlier datasets, which could not be revealed in previous studies utilizing mainly neutral markers. In this study, different cluster analyses revealed that Hilsa shad individuals collected from nine geographical locations are divided into two genetic clusters, in which one cluster is dominated by the marine (deep sea, DS; shallow sea, SS) and estuarine (Meghna estuary, ME) water individuals and another cluster is dominated by the different freshwater riverine (MR, Meghna river; SKR, Surma–Kushiara river; LPR, lower Padma river; UPR, upper Padma river; LJR, lower Jamuna river; UJR, upper Jamuna river) individuals. The distinction between these two major clusters indicates that the gene flow among them is restricted due to a limited dispersal and selection processes, which operate at various scales throughout the heterogeneous environment [53] and likely influences their divergence. More elaborately, such discrimination of Hilsa shad individuals from different geographical locations divided into two genetic clusters can be interpreted by two different ways. Firstly, the genetic discrimination may correspond to the alternative multi-locus genotypes that are maintained by strong divergent selection pressures in a spatially heterogeneous environment in which many factors could impose selection pressures, and those selection pressures could act at one or more life history stages [53]. Secondly, the observed two genetic clusters may correspond to the intense spawning site fidelity for two geographically isolated spawning grounds, one in the freshwater habitats and another one in the brackish water estuary as reported by the previous studies [5,6,7].

The STRUCTURE analysis of the outlier loci demonstrated that individuals from the various locations were assigned either entirely to one subpopulation or entirely to the other subpopulation, indicating that the admixture between these two genetically distinct ecotypes is very limited and may also be reproductively isolated from each other (see the Figure 7). The Hilsa shad individuals collected from the upper stream sites, such as UJR, is highly differentiated from those of the marine individuals collected from DS, with a low possibility that the adult Hilsa spawns in the freshwater environment of the north of Bangladesh and migrates to the deep sea. In our previous study, we showed that the juveniles of Hilsa shad (locally known as *Jatka*) of a particular habitat return to their respective natal rivers (the rivers where they were born/nursed) for spawning as adults [59]. It is also possible that the marine subtype only inhabits the marine environment and relies on the estuarine waters in the Meghna estuary (ME) for spawning, without migrating to the freshwater system [5,6,7]. Another possibility is that the freshwater fish, also known as potamodromous subtypes, rely on the freshwater rivers for spawning and remain and complete their life cycle within the freshwater, without migrating to the sea [5,6,7]. However, according to the pairwise F_ST_ values (Table 2), SS is likely the main foraging ground and acts as the main hub for gene flow exchange. This is evident from the relatively homogenized genetic structure between the Hilsa shad from the SS collection site with other freshwater collection sites in the lower part of the river system (MR, SKR, LJR, LPR), with an FST range of 0.050 (*p* ≤ 0.01) to 0.127 (*p* ≤ 0.001). Accordingly, the Hilsa shad individuals from similar tributaries share similar structuring patterns, which evidently supports the isolation by distance (IBD) concept and the ecologically adaptive behavior of the Hilsa shad. This species spends most of its lifetime in the shallow sea (SS), becomes sexually mature, and migrates to its respective natal spawning grounds in the upper river systems [60]. This migration behavior is compatible with the movements of Hilsa shad individuals at different stages between reproductive areas (LJR, LPR, MR, UJR, UPR, and SKR) and foraging grounds (ME, SS, and DS), which essentially shapes the population structure and is involved in the adaptive divergence of Hilsa shad, like those in other migratory fish species [61]. It is most likely that the selective constraints and local adaptation in the natal spawning areas overcame the genetically homogenizing effects of the neutral loci due to the sufficient gene flow exchange between the populations. 

The NJ phylogenetic trees, based on the outlier loci detected by the two approaches, further subdivided the major riverine Hilsa shad clusters into two discrete sub-clusters: The north-eastern and north-western riverine groups. Therefore, the such structuring into two sub-clusters, which mirrors the spatial structuring that is maintained by environmental differences, such as the turbidity and salinity levels in the marine or brackish, muddy/turbid freshwater (north-western riverine) and clear freshwater (north-eastern riverine) habitats. Due to the effect of the different environmental conditions, the fishes of the north-western riverine habitats are of a bright silvery color, with a thicker structure, while the fishes of the north-eastern riverine are slimmer, a bit darker and elongated [62]. Previous meristic and morphometric studies have also revealed some morphological differences among the Hilsa stocks from different environments [63]. Owing to the distinctive differences, the Hilsa stocks were divided into the north-western riverine’s “broad type” and the north-eastern riverine’s “slender type” [64,65]. The differential seasonal spawning and fecundity level displayed by these morphotypes—the “broad type” during the late monsoon and “slender type” during the monsoon—may have also contributed to the limited natal and breeding dispersal, leading to the divergence of the freshwater Hilsa shad populations [66]. Besides the environmental differences among the collection sites, the clustering pattern of north-eastern and north-western riverine groups may also be related to the spatial autocorrelation of genotype frequencies and observed the IBD pattern of the Hilsa shad individuals [67,68].

The ability to detect a population divergence at the above scales for an anadromous species, like the Hilsa shad, may also depend on the number of outlier loci used. In this study, we employed two independent detection methods for the outliers, notably the F_ST_ OutFLANK and pcadapt approaches, which successfully identified 74 and 449 SNPs, respectively (Figure 2). While utilizing multiple methods for outlier loci detection, followed by intersecting the results, limits the discovery of candidate adaptive markers, which represent a more conservative approach that reduces Type I errors due to false positives [69]. All of the above-mentioned multivariate and individual-based analyses have shown that a reduction in the number of outlier loci greatly increased the signal of divergence, which divided the Hilsa shad individual from different collection sites into two ecotypes. Notably, the F_ST_ OutFLANK dataset outperformed pcadapt in defining the clustering pattern, with a greater confidence level. The identification of 474 putatively adaptive loci using these two different outlier tests supports the notion that they are under selective differentiation across the sampled locations, although some of the observed patterns may still be affected by IBD [67,68]. Given the migratory nature of Hilsa shad, as an anadromous species, this clearer separation substantiated the notion that the genetic divergences between the Hilsa populations are caused by both IBD and dispersion processes. 

The outlier loci also provided a selection of genomic regions associated with the ecotype divergence over recent temporal scales [67]. Of the 74 and 449 SNPs detected by the F_ST_ OutFLANK and pcadapt approaches, respectively, only 35 and 11 SNPs were successfully annotated through BLAST analysis, while a total of 10 outlier loci with known functions were shared by both approaches (Figure 3B). Only 6.8% of the outliers detected by both methods were mapped to the known sequence in the public database, which; however, demonstrated the limitation of RAD sequencing in identifying candidate genes without a reference genome [70]. Previous studies have also reported that local adaptation in fish populations is related to the presence of linked alleles, associated with life history traits [71], which is important in defining the structuring pattern of Hilsa shad. By focusing on genes related to environmental conditions, we found that about five prominent genes (nrxn3b, TRIM67, Grik2, GABBR1, and Herc1) involved in the genetic differences are those related to neuronal development (Table 4). The potential role of the neuron system suggests that adaptive differences may be behavioral in nature. In support of this, nrxn3b, TRIM67, Grik2, GABBR1, and Herc1 are thought to be important for spatial memory, muscle function, and sensory motor development in vertebrates [72,73,74]. We hypothesize that the learning and memory storage ability of Hilsa shad may assist in their migratory behavior by allowing them to recognize their natal spawning ground for breeding, when they become sexually mature. It also seems to be highly possible that habitat differences promote adaptive divergence among Hilsa populations, particularly at early developmental stages. Our BLAST results show that the outlier loci (SCED01000696.1:30199 and SCED01008207.1:36816) are associated with two genes, namely, KDM6A and UBR3, which play a direct role in growth and embryonic development processes. These genes are regulated by various environmental factors, including salinity, oxygen, temperature, latitude, depth and habitat, during the development of an animal species [39]. Furthermore, many genes (SCED01077141.1:127, SCED01094462.1:170, SCED01011615.1:5090, SCED01000975.1:25910, etc.) that encode putatively adaptive loci are involved in metabolism (particularly in glucose, glycogen, and lipid metabolism) to provide sufficient energy during migration. The identified set of genes and multiple processes that are significantly associated with the migratory behavior of Hilsa in this study will be extremely useful in generating hypotheses in future targeted research into unraveling the mechanisms of selection.

## 5. Conclusions

The present study has provided an unprecedented solution to the stock structure of anadromous Hilsa shad populations across nationwide geographical gradients to obtain their special distribution pattern in Bangladesh. This study has also identified isolation by distance patterns, and the NextRAD sequencing has revealed a highly significant discrimination of Hilsa shad populations. Different spatial analyses subdivided Hilsa shad individuals collected from nine strategic geographical locations into two main clusters, one cluster was mostly dominated by the southern marine–estuarine individuals, while another one was mostly dominated by the northern riverine groups, although somewhat heterogenous mixture of both genetic stocks were evident in most of the collection sites due to their anadromous natures. Moreover, our results have confirmed that the northern riverine Hilsa shad population is further sub-clustered into the north-eastern (clear freshwater) and north-western (turbid freshwater) riverine groups. Besides the environmental differences, these genetic discrimination of Hilsa shad population might also be related to spatial autocorrelation of genotype frequencies and the IBD pattern. Our study provides both a neutral and adaptive context for the observed genetic divergence of Hilsa shad and, consequently, reconciles the previous inconclusive findings on their population genetic structure across their diverse migratory habitats. In the context of fisheries management, our results suggest that it is important to maintain the genetic diversity of Hilsa shad in each habitat, as the fishes are capable of a high level of gene flow within the population but a lower variation across most of their migratory distribution range. Local adaptation due to the imposing selective pressure has a pivotal role in maintaining high frequencies of particular genetic traits in each heterogenic habitat. Therefore, special attention should be paid to the management of the upstream riverine Hilsa shad population, given that the selective pressures that maintain the adaptive genetic variations in these habitats are at a high risk due to increased fishing pressure and adverse climatic conditions. Ultimately, our study has clearly demonstrated that NextRAD sequencing is an innovative approach to exploring how dispersal and local adaptation can shape the genetic divergence of non-model anadromous fish that are found in diverse migratory habitats during their life-history stages.

## Figures and Tables

**Table 1:** Summary of the sampling information on *Tenualosa ilisha* for NextRAD sequencing.

Collection Sites	Migratory Habitat	Year of Collection	Latitude	Longitude	Sample Size
DS	Deep sea (Bay of Bengal)	2018	21.2722	91.0632	30
SS	Shallow sea (Bay of Bengal)	2017	21.7927	90.1120	30
ME	Meghna estuary	2017	22.0482	90.9188	30
MR	Meghna river	2018	23.0805	90.6413	30
SKR	Surma–Kushiara river	2018	24.5794	91.2256	30
LPR	Lower Padma river	2018	23.3117	90.5208	30
UPR	Upper Padma river	2017	24.6338	88.0582	60
LJR	Lower Jamuna river	2018	25.1046	89.6869	30
UJR	Upper Jamuna river	2017	25.5138	89.6827	30

DS, deep sea; SS, shallow sea; ME, Meghna estuary; MR, Meghna river; SKR, Surma–Kushiara river; LPR, lower Padma river; UPR, upper Padma river; LJR, lower Jamuna river; UJR, upper Jamuna river.

**Table 2:** Pairwise F_ST_ values for the putatively neutral (above diagonal) and putatively adaptive (below diagonal) panel of SNP loci of *T. ilisha* collected from the nine strategic collection sites, identified by the F_ST_ OutFLANK and pcadapt approaches.

Habitats	DS	LJR	MR	LPR	ME	SKR	SS	UJR	UPR
	*F_ST_ OutFLANK*								
**DS**	--	0.002 ***	0.002 ***	0.002 ***	0.002 ***	0.003 ***	0.001 ***	0.003 ***	0.001 ***
**LJR**	0.230 ***	--	0.003 ***	0.003 ***	0.003 ***	0.004 ***	0.002 ***	0.003 ***	0.002 ***
**MR**	0.138 ***	0.160 ***	--	0.003 ***	0.001 **	0.003 ***	0.001 ***	0.004 ***	0.003 ***
**LPR**	0.145 ***	0.028 *	0.076 **	--	0.002 ***	0.004 ***	0.001 ***	0.003 ***	0.002 ***
**ME**	0.010 ^NS^	0.238 ***	0.161 ***	0.152 ***	--	0.004 ***	0.001 **	0.003 ***	0.003 ***
**SKR**	0.173 ***	0.169 ***	0.030 *	0.106 ***	0.195 ***	--	0.002 ***	0.004 ***	0.003 ***
**SS**	0.020 *	0.127 ***	0.064 **	0.050 **	0.028 *	0.095 ***	--	0.002 ***	0.001 ***
**UJR**	0.315 ***	0.020 *	0.223 ***	0.067 ***	0.327 ***	0.211 ***	0.197 ***	--	0.002 ***
**UPR**	0.206 ***	0.012 ^NS^	0.159 ***	0.034 *	0.212 ***	0.150 ***	0.109 ***	0.031 **	--
	*pcadapt*								
**DS**	--	0.002 ***	0.002 ***	0.002 ***	0.002 ***	0.004 ***	0.001 ***	0.003 ***	0.001 ***
**LJR**	0.052 ***	--	0.003 ***	0.003 ***	0.003 ***	0.005 ***	0.002 ***	0.003 ***	0.002 ***
**MR**	0.030 ***	0.011 **	--	0.003 ***	0.002 ***	0.004 ***	0.002 ***	0.004 ***	0.003 ***
**LPR**	0.036 ***	0.002 ^NS^	0.007 *	--	0.002 ***	0.005 ***	0.001 ***	0.003 ***	0.002 ***
**ME**	0.003 ^NS^	0.053 ***	0.029 ***	0.038 ***	--	0.004 ***	0.001 ***	0.003 ***	0.003 ***
**SKR**	0.032 ***	0.007 *	0.001 ^NS^	0.003 ^NS^	0.036 ***	--	0.002 ***	0.005 ***	0.003 ***
**SS**	0.007 *	0.023 **	0.005 ^NS^	0.012 **	0.007 *	0.009 *	--	0.002 ***	0.001 ***
**UJR**	0.070 ***	0.003 ^NS^	0.020 ***	0.010 ***	0.073 ***	0.013 ***	0.035 ***	--	0.002 ***
**UPR**	0.042 ***	0.001 ^NS^	0.008 **	0.001 ^NS^	0.045 ***	0.005 *	0.016 ***	0.004 *	--

DS, deep sea; SS, shallow sea; ME, Meghna estuary; MR, Meghna river; SKR, Surma–Kushiara river; LPR, lower Padma river; UPR, upper Padma river; LJR, lower Jamuna river; UJR, upper Jamuna river. NS, not significant, * *p* ≤ 0.05, ** *p* ≤ 0.01, *** *p* ≤ 0.001.

**Table 3:** Molecular analysis of variance for the putatively neutral and adaptive panel of SNP loci within and among the different collection sites of *T. ilisha* identified by the F_ST_ OutFLANK and pcadapt approaches.

Loci Category	Source of Variation	Sum of Square	Variance Components	% of Variation	Statistics	*p*-Value
Adaptive panel of SNP loci	*F_ST_ OutFLANK*					
	Within individuals	3468	11.56	103.1	F_it = −0.031	-
	Among individuals	2529.7	−1.433	−12.8	F_is = −0.142	1.00
	Among collection sites	645.337	1.091	9.7	F_st = 0.097	0.000
	*pcadapt*					
	Within individuals	17,112.5	57.042	101.7	F_it = −0.017	-
	Among individuals	15,559.02	−1.787	−3.2	F_is = −0.032	1.00
	Among collection sites	871.202	0.84	1.5	F_st = 0.015	0.000
Neutral panel of SNP loci	*F_ST_ OutFLANK*					
	Within individuals	599,152.5	1997.175	100.5	F_it = −0.005	-
	Among individuals	573,245.4	−13.63	−0.7	F_is = −0.007	1.00
	Among collection sites	17,503.28	3.303	0.2	F_st = 0.002	0.000
	*pcadapt*					
	Within individuals	585,923	1953.077	100.5	F_it = −0.005	-
	Among individuals	560,559.6	−13.377	−0.7	F_is = −0.007	1.00
	Among collection sites	17,326.43	3.629	0.2	F_st = 0.002	0.000

**Table 4:** Summary of the gene functions and annotation based on the reference genomes available in the public database of the putatively adaptive panel of the SNP loci of *T. ilisha* populations.

Locus Name	GenBank Accession Number	Gene Name ^1^	Species	Biological Function ^2^
SCED01086260.1:268	XM_012815555.1	nrxn3b *	*Clupea harengus*	Neuronal cell surface protein
SCED01010228.1:165	XM_012823864.1	TRIM67 *	*C. harengus*	Neuron projection development
SCED01084234.1:74	XM_012823924.1	TTC13 *	*C. harengus*	Unknown
SCED01037310.1:1343	XM_012823959.1	MTHFD1 *	*C. harengus*	Somite and heart development
SCED01066042.1:124	XM_012828951.1	Grik2 *	*C. harengus*	Neuronal activity
SCED01072826.1:68	XM_012835569.1	EXTL3 *	*C. harengus*	Biosynthesis of polysaccharides
SCED01077141.1:127	XM_012839387.1	PLCB1 *	*C. harengus*	Lipid degradation and metabolism
SCED01094462.1:170	XM_017701045.1	ITPKB *	*Pygocentrus nattereri *	Metabolic process, ATP binding
SCED01094733.1:323	XM_023923858.1	EXOC8 *	*Cyanistes caeruleus*	Exocytosis and protein transport
SCED01029258.1:4937	XM_028970071.1	NFIB *	*Denticeps clupeoides*	Differentiation, DNA replication
SCED01007742.1:237924	XM_012823803.1	FBXO11 ^a^	*C. harengus*	Cellular protein modification
SCED01001444.1:144867	XM_008399996.2	PRLHR	*Poecilia reticulata*	Feeding behavior, metabolic process
SCED01000736.1:64807	XM_012815189.1	U2SURP	*C. harengus*	mRNA splicing
SCED01012000.1:38243	XM_012822717.1	MYO6	*C. harengus*	Movement, endocytosis
SCED01013770.1:62161	XM_012824084.1	Gmps	*C. harengus*	Guanosine monophosphate biosynthesis
SCED01008867.1:80237	XM_012827367.1	EPR1	*C. harengus*	Cellular membrane biosynthesis
SCED01003175.1:15453	XM_012827500.1	Elfn2	*C. harengus*	Protein phosphatase inhibitor
SCED01030760.1:318	XM_012828774.1	SLC7A10	*C. harengus*	Amino acids transport
SCED01009040.1:131462	XM_012828861.1	BLMH	*C. harengus*	Response to toxic substances
SCED01011615.1:5090	XM_012830641.1	AADAT	*C. harengus*	Biosynthetic and metabolic process
SCED01010819.1:27239	XM_012830963.1	SIDT2	*C. harengus*	Glucose homeostasis
SCED01020677.1:12909	XM_012832078.1	MYRF	*C. harengus*	Differentiation and transcription
SCED01010957.1:39086	XM_012832114.1	SFI1	*C. harengus*	Mitotic cell cycle regulation
SCED01005892.1:34793	XM_012832603.1	GABBR1	*C. harengus*	Neuronal cell signaling
SCED01000975.1:25910	XM_012833200.1	PDHA1	*C. harengus*	Glucose metabolic process
SCED01010852.1:64989	XM_012835295.1	ACIN1	*C. harengus*	Apoptosis and mRNA processing
SCED01002831.1:133084	XM_012837338.1	TUBGCP6	*C. harengus*	Microtubule nucleation
SCED01000696.1:30199	XM_012837568.1	KDM6A	*C. harengus*	Embryonic development
SCED01003627.1:221050	XM_012838801.1	CCNF	*C. harengus*	Mitotic cell division control
SCED01010418.1:19123	XM_012840278.1	Herc1	*C. harengus*	Neuromuscular process
SCED01024554.1:1059	XM_012841400.1	ARAP1	*C. harengus*	GTPase activity
SCED01022525.1:8702	XM_012841845.1	PPP1R9B	*C. harengus*	Cell cycle control and neurogenesis
SCED01000852.1:137973	XM_012842171.1	PGM1	*C. harengus*	Synthesis glucose and glycogen
SCED01062845.1:131	XM_015395566.1	MPEG1	*Cyprinodon variegatus*	Immune response against bacteria
SCED01008207.1:36816	XM_026272763.1	UBR3	*Carassius auratus*	Embryonic development
SCED01003543.1:101682	XR_001162390.1	SRP68	*C. harengus*	Role in targeting secreting protein

^1^ Gene names are abbreviated following the UniPort database (https://www.uniprot.org). For details of the gene names, please see the supplementary Microsoft excel file. ^2^ Only the most important biological functions of the specific genes in vertebrates from UniPort database are reported. Genes marked with an * are putatively SNP loci, identified by both the F_ST_ OutFLANK and pcadapt approaches; genes marked with an ^a^ are putatively SNP loci, identified by the F_ST_ OutFLANK approach only, and the remaining unmarked genes are putatively SNP loci, identified by the pcadapt approach only.

## Abbreviations

NGS	Next-generation sequencing
NextRAD	NGS-based restriction-site-associated DNA
RAD-seq	Restriction site-associated DNA sequencing
DS	Deep sea
SS	Shallow sea
ME	Meghna estuary
MR	Meghna river
SKR	Surma–Kushiara river
LPR	Lower Padma river
UPR	Upper Padma river
LJR	Lower Jamuna river
UJR	Upper Jamuna river
AMOVA	Analysis of molecular variance
PCA	Principal component analysis
DAPCs	Discriminant analysis of principal components
NJ	Neighbor-joining
QDFA	Quadratic discriminant functional analysis
SNP	Single nucleotide polymorphism

## References

[1] Islam M.M., Essam Y.M., Ali L. (2016). Economic incentives for sustainable Hilsa fishing in Bangladesh: An analysis of the legal and institutional framework. Mar. Policy.

[2] Rahman M.J., Wahab M.A., Amin S.M.N., Nahiduzzaman M., Romano N. (2018). Catch Trend and Stock Assessment of Hilsa *Tenualosa ilisha* Using Digital Image Measured Length-Frequency Data. Mar. Coast. Fish..

[3] DOF (Department of Fisheries) (2018). Yearbook of Fisheries Statistics of Bangladesh 2017–18.

[4] Bernatchez L. (2016). On the maintenance of genetic variation and adaptation to environmental change: Considerations from population genomics in fishes. J. Fish Biol..

[5] Blaber S.J.M., Milton D.A., Chenery S.R., Fry G.C. (2003). New insights into the life history of *Tenualosa ilisha* and fishery implications. Am. Fish. Soc. Symp..

[6] Milton D.A., Chenery S.R. (2001). Can Otolith chemistry detect the population structure of the shad hilsa *Tenualosa ilisha*? Comparison with genetic and morphological studies. Mar. Ecol. Prog. Ser..

[7] Hossain M.S., Sarker S., Sharifuzzaman S.M., Chowdhury S.R. (2014). Discovering spawning ground of Hilsa Shad (*Tenualosa ilisha*) in the coastal waters of Bangladesh. Ecol. Model..

[8] Hess J.E., Campbell N.R., Close D.A., Docker M.F., Narum S.R. (2012). Population genomics of Pacific lamprey: Adaptive variation in a highly dispersive species. Mol. Ecol..

[9] Allendorf F.W., Hohenlohe P.A., Luikart G. (2010). Genomics and the future of conservation genetics. Nat. Rev. Genet..

[10] Carreras C., Ordóñez V., Zane L., Kruschel C., Nasto I., Macpherson E., Pascual M. (2016). Population genomics of an endemic Mediterranean fish: Differentiation by fine scale dispersal and adaptation. Sci. Rep..

[11] Drinan D.P., Gruenthal K.M., Canino M.F., Lowry D., Fisher M.C., Hauser L. (2018). Population assignment and local adaptation along an isolation-by-distance gradient in Pacific cod (*Gadus macrocephalus*). Evol. Appl..

[12] Wright D., Bishop J.M., Matthee C.A., von der Heyden S. (2015). Genetic isolation by distance reveals restricted dispersal across a range of life histories: Implications for biodiversity conservation planning across highly variable marine environments. Divers. Distrib..

[13] Gamboa M., Watanabe K. (2019). Genome-wide signatures of local adaptation among seven stoneflies species along a nationwide latitudinal gradient in Japan. BMC Genom..

[14] Rahman M., Naevdal G. (2000). Population genetics studies of Hilsa shad, *Tenualosa ilisha* (Hamilton), in Bangladesh waters: Evidence for the existence of separate gene pools. Fish. Manag. Ecol..

[15] Salini J.P., Milton D.A., Rahman M.J., Hussain M.G. (2004). Allozyme and morphological variation throughout the geographic range of the tropical shad, Hilsa *Tenualosa ilisha*. Fish. Res..

[16] Ahmed A.S.I., Islam M.S., Azam M.S., Khan M.M.R., Alam M.S. (2004). RFLP analysis of the mtDNA D-loop region in Hilsa shad (*Tenualosa ilisha*) population from Bangladesh. Indian J. Fish.

[17] Mazumder S.K., Alam M.S. (2009). High levels of genetic variability and differentiation in Hilsa shad, *Tenualosa ilisha* (Clupeidae, Clupeiformes) populations revealed by PCR-RFLP analysis of the mitochondrial DNA D-loop region. Genet. Mol. Biol..

[18] Brahmane M.P., Kundu S.N., Das M.K., Sharma A.P. (2013). Low genetic diversity and absence of population differentiation of Hilsa (*Tenualosa ilisha*) revealed by mitochondrial DNA cytochrome b region in Ganga and Hooghly rivers. Afr. J. Biotechnol..

[19] Behera B.K., Singh N.S., Paria P., Sahoo A.K., Panda D., Meena D.K., Das P., Pakrashi S., Biswas D.K., Sharma A.P. (2015). Population genetic structure of Indian shad, *Tenualosa ilisha* Inferred from variation in mitochondrial DNA sequences. J. Environ. Biol..

[20] Hemmer-Hansen J., Nielsen E.E., Frydenberg J., Loeschcke V. (2007). Adaptive divergence in a high gene flow environment: Hsc70 variation in the European flounder (*Platichthys flesus* L.). Heredity.

[21] Schunter C., Garza J.C., Macpherson E., Pascual M. (2014). SNP development from RNA-seq data in a nonmodel fish: How many individuals are needed for accurate allele frequency prediction?. Mol. Ecol. Resour..

[22] Andrews K.R., Good J.M., Miller M.R., Luikart G., Hohenlohe P.A. (2016). Harnessing the power of RADseq for ecological and evolutionary genomics. Nat. Rev. Genet..

[23] Star B., Nederbragt A.J., Jentoft S., Grimholt U., Malmstrom M., Gregers T.F., Rounge T.B., Paulsen J., Solbakken M.H., Sharma A. (2011). The genome sequence of Atlantic cod reveals a unique immune system. Nature.

[24] Barson N.J., Aykanat T., Hindar K., Baranski M., Bolstad G.H., Fiske P., Jacq C., Jensen A.J., Johnston S.E., Karlsson S. (2015). Sex-dependent dominance at a single locus maintains variation in age at maturity in salmon. Nature.

[25] Hohenlohe P.A., Bassham S., Etter P.D., Stiffler N., Johnson E.A., Cresko W.A. (2010). Population genomics of parallel adaptation in threespine stickleback using sequenced RAD tags. PLoS Genet..

[26] Bélanger-Deschênes S., Couture P., Campbell P.G.C., Bernatchez L. (2013). Evolutionary change driven by metal exposure as revealed by coding SNP genome scan in wild yellow perch (*Perca flavescens*). Ecotoxicology.

[27] Laporte M., Rogers S.M., Dion-Cote A.M., Normandeau E., Gagnaire P.A., Dalziel A.C., Chebib J., Bernatchez L. (2015). RAD-QTL Mapping reveals both genome-level parallelism and different genetic architecture underlying the evolution of body shape in lake whitefish (*Coregonus clupeaformis*) species pairs. G3: Genes Genom. Genet..

[28] Hauser L., Carvalho G.R. (2008). Paradigm shifts in marine fisheries genetics: Ugly hypotheses slain by beautiful facts. Fish Fish..

[29] Bradbury I.R., Hubert S., Higgins B., Borza T., Bowman S., Paterson I.G., Snelgrove P.V.R., Morris C.J., Gregory R.S., Hardie D.C. (2010). Parallel adaptive evolution of Atlantic cod on both sides of the Atlantic Ocean in response to temperature. Proc. R. Soc. B.

[30] Lamichhaney S., Barrio A.M., Rafati N., Sundström G., Rubin C.J., Gilbert E.R., Berglund J., Wetterbom A., Laikre L., Grabherr M. (2012). Population-scale sequencing reveals genetic differentiation due to local adaptation in Atlantic herring. Proc. Natl. Acad. Sci. USA.

[31] Ogden R., Gharbi K., Mugue N., Martinsohn J., Senn H., Davey J.W., Pourkazemi M., McEwing R., Eland C., Vidotto M. (2013). Sturgeon conservation genomics: SNP discovery and validation using RAD sequencing. Mol. Ecol..

[32] Benestan L., Gosselin T., Perrier C., Sainte-Marie B., Rochette R., Bernatchez L. (2015). RAD genotyping reveals fine-scale genetic structuring and provides powerful population assignment in a widely distributed marine species, the American lobster (*Homarus americanus*). Mol. Ecol..

[33] Baird N.A., Etter P.D., Atwood T.S., Currey M.C., Shiver A.L., Lewis Z.A., Johnson E.A. (2008). Rapid SNP Discovery and Genetic Mapping Using Sequenced RAD Markers. PLoS ONE.

[34] Elshire R.J., Glaubitz J.C., Sun Q., Poland J.A., Kawamoto K., Buckler E.S., Mitchell S.E. (2011). A Robust, Simple Genotyping-by-Sequencing (GBS) Approach for High Diversity Species. PLoS ONE.

[35] Davey J.L., Blaxter M.W. (2010). RADseq: Next-generation population genetics. Brief. Funct. Genom..

[36] Allison M.A. (1998). Geologic Framework and Environmental Status of the Ganges-Brahmaputra Delta. J. Coast. Res..

[37] Barua D.K. (1990). Suspended sediment movement in the estuary of the Ganges-Brahmaputra-Meghna River system. Mar. Geol..

[38] Hasan K.M.M., Wahab A., Ahmed Z.F., Mohammed E.Y. (2015). The Biophysical Assessments of the Hilsa Fish (Tenualosa Ilisha) Habitat in the Lower Meghna, Bangladesh.

[39] Gilbert S.F. (2005). Mechanisms for the environmental regulation of gene expression: Ecological aspects of animal development. J. Biosci..

[40] Emerson K.J., Conn J.E., Bergo E.S., Randel M.A., Sallum M.A. (2015). Brazilian Anopheles darlingi root (Diptera: Culicidae) clusters by major biogeographical region. PLoS ONE.

[41] Jombart T. (2008). adegenet: An R package for the multivariate analysis of genetic markers. Bioinformatics.

[42] Whitlock M.C., Lotterhos K.E. (2015). Reliable Detection of Loci Responsible for Local Adaptation: Inference of a Null Model through Trimming the Distribution of F_ST_. Am. Nat..

[43] Luu K., Bazin E., Blum M.G.B. (2017). pcadapt: An R package to perform genome scans for selection based on principal component analysis. Mol. Ecol. Resour..

[44] Duforet-Frebourg N., Luu K., Laval G., Bazin E., Blum M.G. (2015). Detecting genomic signatures of natural selection with principal component analysis: Application to the 1000 Genomes data. Mol. Biol. Evol..

[45] Meirmans P.G., van Tienderen P.H. (2004). GENOTYPE and GENODIVE: Two programs for the analysis of genetic diversity of asexual organisms. Mol. Ecol. Notes.

[46] Pritchard J.K., Stephens M., Donnelly P. (2000). Inference of population structure using multilocus genotype data. Genetics.

[47] Evanno G., Regnaut S., Goudet J. (2005). Detecting the number of clusters of individuals using the software structure: A simulation study. Mol. Ecol..

[48] Earl D.A., vonHoldt B.M. (2012). STRUCTURE HARVESTER: A website and program for visualizing STRUCTURE output and implementing the Evanno method. Conserv. Genet. Resour..

[49] Kopelman N.M., Mayzel J., Jakobsson M., Rosenberg N.A., Mayrose I. (2015). Clumpak: A program for identifying clustering modes and packaging population structure inferences across K. Mol. Ecol. Resour..

[50] Funk W.C., McKay J.K., Hohenlohe P.A., Allendorf F.W. (2012). Harnessing genomics for delineating conservation units. Trends Ecol. Evol..

[51] Milano I., Babbucci M., Cariani A., Atanassova M., Bekkevold D., Carvalho G.R., Espiñeira M., Fiorentino F., Garofalo G., Geffen A.J. (2014). Outlier SNP markers reveal fine-scale genetic structuring across European hake populations *(Merluccius merluccius*). Mol. Ecol..

[52] Hollenbeck C.M., Portnoy D.S., Gold J.R. (2019). Evolution of population structure in an estuarine-dependent marine fish. Ecol. Evol..

[53] Salas E.M., Bernardi G., Berumen M.L., Gaither M.R., Rocha L.A. (2019). RADseq analyses reveal concordant Indian Ocean biogeographic and phylogeographic boundaries in the reef fish *Dascyllus trimaculatus*. R. Soc. Open Sci..

[54] Dahle G., Rahman M., Eriksen A.G. (1997). RAPD finger printing used for discriminating among three populations of Hilsa shad (*Tenualosa ilisha*). Fish. Res..

[55] Shifat R., Begum A., Khan H. (2003). Use of RAPD fingerprinting for discriminating two populations of Hilsa shad (*Tenualosa ilisha* Ham.) from inland rivers of Bangladesh. J. Biochem. Mol. Biol..

[56] Brahmane M.P., Das M.K., Sinha M.R., Sugunan V.V., Mukherjee A., Singh S.N., Prakash S., Maurye P., Hajra A. (2006). Use of RAPD fingerprinting for delineating populations of hilsa shad *Tenualosa ilisha* (Hamilton, 1822). Genet. Mol. Res..

[57] Jorfi E., Farhad A., Ghorezshi S.A., Mortezaei S.R.S. (2008). A study on population genetic of hilsa shad, *Tenualosa iliisha* in Khoozestan, Iran using molecular method (RAPD). Iran. Sci. Fish. J..

[58] Gralka M., Stiewe F., Farrell F., Möbius W., Waclaw B., Hallatschek O. (2016). Allele surfing promotes microbial adaptation from standing variation. Ecol. Lett..

[59] Asaduzzaman M., Wahab M.A., Rahman M.J., Nahiduzzaman M., Dickson M.W., Igarashi Y., Asakawa S., Wong L.L. (2019). Fine-scale population structure and ecotypes of anadromous Hilsa shad (*Tenualosa ilisha*) across complex aquatic ecosystems revealed by NextRAD genotyping. Sci. Rep..

[60] Hossain M.S., Sharifuzzaman S.M., Chowdhury S.R. (2016). Habitats across the life cycle of Hilsa shad (*Tenualosa ilisha*) in aquatic ecosystem of Bangladesh. Fish. Manag. Ecol..

[61] Pecoraro C., Babbucci M., Franch R., Rico C., Papetti C., Chassot E., Bodin N., Cariani A., Bargelloni L., Tinti F. (2018). The population genomics of yellowfin tuna (*Thunnus albacares*) at global geographic scale challenges current stock delineation. Sci. Rep..

[62] Mojumdar C.H. (1939). Culture of Hilsa. Mod. Rev..

[63] Quddus M.M.A., Shimizu M., Nose Y. (1984). Meristic and morphometric differences in two types of *Hilsa ilisha* in Bangladesh waters. Bull. Jpn. Soc. Sci. Fish..

[64] Ghosh A.N., Bhattacharya R.K., Rao K.V. (1968). On the identification of the sub-populations of *Hilsa ilisha* (Ham.) in the Gangetic system with a note on their distribution. Proc. Natl. Acad. Sci. India Sect. B Biol. Sci..

[65] Shafi M., Quddus M.M.A., Hossain H. (1977). A morphometric study of the population of *Hilsa ilisha* (Hamilton-Buchanan) from the river Meghna. Proc. Second Bangladesh Sci. Conf..

[66] Pillay S.R., Rao K.V., Mathur P.K. (1962). Preliminary report on the tagging of the Hilsa, *Hilsa ilisha* (Hamilton). Proc. Indo Pac. Fish. Counc..

[67] Lotterhos K.E., Whitlock M.C. (2014). Evaluation of demographic history and neutral parameterization on the performance of F_ST_ outlier tests. Mol. Ecol..

[68] Meirmans P.G. (2012). The trouble with isolation by distance. Mol. Ecol..

[69] Russello M.A., Kirk S.L., Frazer K.K., Askey P.J. (2012). Detection of outlier loci and their utility for fisheries management. Evol. Appl..

[70] Picq S., McMillan W.O., Puebla O. (2016). Population genomics of local adaptation versus speciation in coral reef fishes (*Hypoplectrus* spp., *Serranidae*). Ecol. Evol..

[71] Barth J.M., Damerau M., Matschiner M., Jentoft S., Hanel R. (2017). Genomic differentiation and demographic histories of Atlantic and Indo-Pacific yellowfin tuna (*Thunnus albacares*) populations. Genome Biol. Evol..

[72] Boyer N.P., Monkiewicz C., Menon S., Moy S.S., Gupton S.L. (2018). Mammalian TRIM67 functions in brain development and behavior. eNeuro.

[73] Schrick C., Fischer A., Srivastava D.P., Tronson N.C., Penzes P., Radulovic J. (2007). N-cadherin regulates cytoskeletally associated IQGAP1/ERK signaling and memory formation. Neuron.

[74] Takahashi N., Sakurai T., Bozdagi-Gunal O., Dorr N.P., Moy J., Krug L., Gama-Sosa M., Elder G.A., Koch R.J., Walker R.H. (2011). Increased expression of receptor phosphotyrosine phosphatase-β/ζ is associated with molecular, cellular, behavioral and cognitive schizophrenia phenotypes. Transl. Psychiatry.

